# Distribution of *Salmonella* Serovars in Humans, Foods, Farm Animals and Environment, Companion and Wildlife Animals in Singapore

**DOI:** 10.3390/ijerph17165774

**Published:** 2020-08-10

**Authors:** Kyaw Thu Aung, Wei Ching Khor, Sophie Octavia, Agnes Ye, Justina Leo, Pei Pei Chan, Georgina Lim, Wai Kwan Wong, Brian Zi Yan Tan, Joergen Schlundt, Anders Dalsgaard, Lee Ching Ng, Yueh Nuo Lin

**Affiliations:** 1 National Centre for Food Science, Singapore Food Agency, Singapore 718837, Singapore; KHOR_Wei_Ching@sfa.gov.sg (W.C.K.); Agnes_YE@sfa.gov.sg (A.Y.); Justina_LEO@sfa.gov.sg (J.L.); 2 Nanyang Technological University Food Technology Centre (NAFTEC), Singapore 637551, Singapore; jschlundt@ntu.edu.sg (J.S.); adal@sund.ku.dk (A.D.); 3 School of Chemical and Biomedical Engineering, Nanyang Technological University, Singapore 637551, Singapore; 4 School of Biological Sciences, Nanyang Technological University, Singapore 637551, Singapore; NG_Lee_Ching@nea.gov.sg; 5 National Public Health Laboratory, National Centre for Infectious Diseases, Singapore 308442, Singapore; Sophie_OCTAVIA@ncid.sg; 6 Ministry of Health, Singapore 169854, Singapore; CHAN_Pei_Pei@moh.gov.sg (P.P.C.); Georgina_LIM@moh.gov.sg (G.L.); 7 Centre for Animal & Veterinary Sciences, Animal and Veterinary Services, National Parks Board, Singapore 718827, Singapore; WONG_Wai_Kwan@nparks.gov.sg (W.K.W.); briantzyan@gmail.com (B.Z.Y.T.); 8 Department of Veterinary and Animal Sciences, University of Copenhagen, 1871 Frederiksberg C, Denmark; 9 Environmental Health Institute, National Environment Agency, Singapore 138667, Singapore; 10 National Centre for Infectious Diseases, Singapore 308442, Singapore; Yueh_Nuo_LIN@ncid.sg

**Keywords:** *Salmonella*, serovar distribution, One-Health, humans, foods, animals

## Abstract

We analyzed the epidemiological distribution of *Salmonella* serovars in humans, foods, animals and the environment as a One-Health step towards identifying risk factors for human salmonellosis. Throughout the 2012–2016 period, *Salmonella* ser. Enteritidis was consistently the predominating serovar attributing to >20.0% of isolates in humans. Other most common serovars in humans include *Salmonella* ser. Stanley, *Salmonella* ser. Weltevreden, *Salmonella* ser. Typhimurium and *Salmonella* ser. 4,5,12:b:-(dT+). *S.* Enteritidis was also the most frequent serovar found among the isolates from chicken/chicken products (28.5%) and eggs/egg products (61.5%) during the same period. In contrast, *S.* Typhimurium (35.2%) and *Salmonella* ser. Derby (18.8%) were prevalent in pork/pork products. *S*. Weltevreden was more frequent in seafood (19.2%) than others (≤3.0%). Most isolates (>80.0%) from farms, companion and wildlife animals belonged to serovars other than *S*. Enteritidis or *S*. Typhimurium. Findings demonstrate the significance of a One-Health investigative approach to understand the epidemiology *Salmonella* for more effective and integrated surveillance systems.

## 1. Introduction

*Salmonella* is one of the major foodborne bacterial pathogens worldwide. It causes gastroenteritis (known as salmonellosis) in humans with clinical manifestations including diarrhea, fever, abdominal cramps, and occasionally invasive infection in humans. It can be typically acquired through consumption of contaminated food, and infrequently through person-to-person contact and contacts with companion animals and wildlife reservoirs [1,2,3]. *Salmonella enterica* represents the most pathogenic species and there are >2600 serovars identified with some showing restricted host selectivity, whereas vast majority of the serovars have broad host spectrum and pose public health risks and can also infect a broad range of animals [2].

In Singapore, the incidence of non-typhoidal salmonellosis has been showing a steadily rising trend, especially since 2008 when the mandatory reporting requirement of salmonellosis was implemented [4]. In 2016, the reported incidence rate of non-typhoidal salmonellosis (39.4 per 100,000 population) was approximately three times higher than that reported in 2008 (14.8 per 100,000 population) [4]. Salmonellosis cases continued to show an uptrend with a significant rise from 2018 (334 cumulative cases at first 13 weeks) to 2019 (536 cumulative cases at first 13 weeks) [5]. Identification of the important serovars, sources and risk factors potentially associated with the infection is important to prioritize food safety and public health measures.

Raw poultry and eggs are generally regarded as the most common vehicles of *Salmonella* [6,7]. In Singapore, the estimated occurrence of *Salmonella,* ranging from 2.7% to 41.3%, was reported in various types of poultry meat across the food chain. While *Salmonella* was rarely found in retail food (0.08%, 17/21,428), the majority of cooked or ready-to-eat food samples detected with *Salmonella* contained poultry meat or eggs [5,8]. The current knowledge therefore suggests poultry and eggs as relatively high-risk ingredients for *Salmonella* contamination. The risk can be further increased by improper hygiene practices at retails such as suboptimal storage conditions, improper heat treatment, post-cooking handling and cross-contamination.

In addition to raw poultry and eggs, other food products such as raw meat and raw seafood can serve as alternative vehicles of *Salmonella* [9,10]. Continued analysis of *Salmonella* serovars over time is important to describe the trends in distribution of *Salmonella* in various ecological sectors in order to identify their relative importance to human health.

To better understand the epidemiology of *Salmonella*, this retrospective study aimed to analyze and describe the distribution of *Salmonella* serovars isolated from humans, foods, farm animals and environment, companion and wildlife animals samples in Singapore. Findings from this multi-agencies’ collaborative study would offer useful insights to enhance our understanding on the epidemiology of *Salmonella* for more effective and integrated surveillance systems.

## 2. Materials and Methods

Data on *Salmonella* isolates obtained during a 5-year study period of 2012 to 2016 were collated from Singapore’s national reference laboratories for public health, food safety, animal health and environmental health: National Public Health Laboratory (NPHL), National Centre for Food Science (NCFS) (former Veterinary Public Health Centre (VPHC)), Centre for Animal & Veterinary Sciences (CAVS) (former Animal Health Laboratory) and Environmental Health Institute (EHI) respectively.

A total of 8004 isolates obtained from humans (4462), foods (2529), chicken and quail layer farms (945), and companion and wildlife animal (68) samples were included for analysis. Sample categories are as shown in Table 1. Human isolates were submitted to NPHL under the Infectious Disease Act (Singapore) by public hospital laboratories. Food isolates were obtained through routine surveillance and monitoring programs, with food samples collected from points of import, wholesalers, slaughterhouses, local produce and food processing establishments in Singapore. The food samples were imported as live, chilled, frozen, or in processed forms. Isolates from farms (chicken and quail layers), companion and wildlife animals were obtained from samples submitted for routine animal health surveillance and monitoring programs or for laboratory diagnostic testing.

All human isolates confirmed to be *Salmonella* spp. by matrix-assisted laser desorption ionization-time of flight mass spectrometry (MALDI-TOF-MS, Bruker, MA, USA) were subjected to serotyping according to the Kauffman-White scheme [11]. PCR and sequencing of flagellin genes *fliB* and *fliC* were carried out to determine phase variation. Biochemical testing and PCR were performed for differentiation of d-tartrate fermentation. For food isolates, *Salmonella* spp. was isolated from 25 g of raw food sample following enrichment in buffered peptone water. Shell eggs were screened serologically for *Salmonella* spp. antibodies and positive consignments were then tested for the presence of *S.* Enteritidis only. Pathogen identification was performed using the Vitek 2 (BioMérieux, Marcy-l’Étoile, France) biochemical system. All food isolates obtained were serotyped by the slide agglutination method according to the Kauffman-White scheme [11]. Samples from farms, companion and wildlife animals were primarily screened for *S.* Enteritidis and *S.* Typhimurium as the target serovars of the different surveillance programs.

Descriptive epidemiological analysis was conducted to identify the most frequently reported serovars in various sample categories. Their significance to public health was evaluated in comparison to the serovars found in human isolates.

## 3. Results

### 3.1. Human Isolates

Since 2012, *S*. Enteritidis was the predominant serovar attributing to >20.0% of all *Salmonella* isolates in humans (Table 2). *S*. Stanley was among the top five serovars detected in humans since 2012. *S*. Weltevreden ranked third (2012–2014) and second (2015–2016). *S*. Typhimurium was not among the top three serovars in the years studied. The monophasic *Salmonella enterica* serovar 4,5,12:b:−(dT+) was ranked fourth (2012) and fifth (2013–2016). *Salmonella* ser. Saintpaul and *Salmonella* ser. Brancaster were reported for the first time among the top 10 prevalent serovars in 2015 and 2016, respectively (Table 2).

### 3.2. Food Isolates

Overall, distribution patterns of dominant serovars in foods were relatively more heterogeneous than that in human cases (Table 2). Analysis of serovar occurrence in various food commodities showed that *S.* Enteritidis (28.5%) and *Salmonella* ser. Heidelberg (21.7%) were the two predominant serovars found in chicken and chicken products (Table 3).

*S.* Typhimurium was the main serovar in duck (43.9%) and poultry other than chicken (21.0%). Similarly, isolates from pork and pork products were dominated by *S.* Typhimurium (35.2%), followed by *S.* Derby (18.8%). *S.* Enteritidis and *S.* Typhimurium were more frequently isolated from fresh than frozen chicken meat (Table 4). *S*. Heidelberg was the predominant serovar in frozen chicken meat except in 2015 (ranked 3rd), and this serovar was not among the top five serovars in fresh chicken.

Similar to human isolates, *S.* Saintpaul and *S.* Brancaster were found to appear among the top 10 prevalent serovars among food samples from 2015. A limited number of isolates obtained from other food products (frog, crocodile, turtle, beef and mutton) represented different serovars (Table 3). Of the 26 isolates obtained from eggs and egg products, 61.5% were identified as *S.* Enteritidis. *S*. Weltevreden was the most frequently found in seafood (19.2%) but less frequently isolated from other food commodities (≤3.0%).

### 3.3. Farm, Companion and Wildlife Animal Isolates

Of the 945 isolates obtained from chicken and quail layer farms, 10.5% and 6.2% were *S.* Enteritidis and *S.* Typhimurium respectively while the majority (>80.0%) of the isolates were non-*S*. Enteritidis and non-*S*. Typhimurium serovars. Similarly, most (>90.0%) of the 68 companion and wildlife animal isolates were identified to be non-*S.* Enteritidis and non-*S.* Typhimurium (Table 3). Serovar level of these non-*S.* Enteritidis and non-*S.* Typhimurium isolates were not identified as the surveillance programs conducted primarily screened for *S.* Enteritidis and *S.* Typhimurium as the target serovars.

## 4. Discussion

In this study, *S.* Enteritidis was found to be the most consistently prevalent serovar associated with human cases since 2012. This is generally in agreement with the global trend of serovars associated with human salmonellosis reported by the US Centers for Diseases Control and Prevention and European Food Safety Authority [12,13]. Global increase in the incidence of *S.* Enteritidis was first noticed in the 1980´s and seems associated with consumption of eggs and poultry [14,15]. *S.* Enteritidis was the most frequent serovar found in chicken and eggs/egg products in our study. In accordance with human isolates, *S.* Enteritidis was consistently found to be the prevalent serovar in both fresh and frozen chicken meat samples from 2012 to 2016. The serovar also appeared among the top ten most frequently isolated serovars in other sample categories, such as duck, seafood, and other types of poultry meat, although at relatively lower frequencies. Compared with companion and wildlife animal samples (1.5%), the serovar was more frequently found in chicken and quail layer farm samples (10.5%).

Chicken is the most common type of meat consumed by the Singaporean population at approximately 34 kg of chicken per capita annually, compared to pork (22 kg), duck (2 kg), mutton (2 kg) and beef (3 kg) [16]. Further, each person consumes an average of 358 eggs annually, a food category where *S.* Enteritidis was the most frequently detected serovar (61.5% of the isolates from eggs and egg products were *S.* Enteritidis) in this study. However, it should be noted that the total number of eggs and egg products tested and analyzed accounted for only 1.0% of the total number of food samples analyzed. Sampling of domestic and imported eggs and egg products should be increased to generate further information about the relative importance of these different products to human salmonellosis. Finding of *S*. Enteritidis being the most frequently detected among the isolates from chicken/chicken products and eggs/egg products reiterates the importance of proper handling and thorough cooking, or to use pasteurized eggs/egg products for food requiring raw or lightly cooked eggs [17]. Further assessment, for instance, risk assessment and source attribution modelling can be applied to estimate the relative contribution of different food categories to human salmonellosis for prioritization of effective intervention strategies in Singapore [6].

Since 2012, there has been an overall downward trend in the relative occurrence of *S.* Enteritidis in food products in Singapore. We surmise that this reduction could be related to improving safety standards of imported foods as a result of Singapore’s regulatory measures on *Salmonella* contamination, particularly upon *S.* Enteritidis detection. These measures include deterring the affected batches from sale, imposing administrative requirements involving targeted testing of potentially affected products, and possible loss of accreditation status for the source farm. Nevertheless, *S.* Enteritidis remains the predominant serovar associated with sporadic and suspected outbreak cases of human salmonellosis in Singapore [5,18]. Further investigations using genomic tools are recommended to describe the lineages of *S*. Enteritidis across sectors to determine the transmission pathway of the serovar.

In recent years, *S.* Typhimurium has been reported as a dominant serovar in humans in European countries where it is often linked to consumption of raw or undercooked pork and pork products [10]. In contrary, our findings showed that *S.* Typhimurium was not among the top three ranked serovars associated with human salmonellosis throughout the study period and less frequently found in clinical samples in 2015 and 2016 when compared with previous years. *S.* Typhimurium was less frequent in chicken and egg products, but the predominant serovar in other foods, i.e., duck, pork and pork products, and other poultry where the serovar accounted for 43.9%, 35.2%, and 21.0% of the isolates, respectively. These findings are in agreement with other studies which reported *S.* Typhimurium as the principal serovar detected in dairy, pork, beef and mutton [16,19]. Detection of *S.* Typhimurium in chicken meat was low (9.4%) however relatively more frequent in fresh than frozen chicken meat. Besides various raw food categories, *S.* Typhimurium was previously found in domestic and wildlife animals in Singapore and elsewhere, suggesting their possible roles in the epidemiology of human salmonellosis [20,21,22].

*S.* Heidelberg (21.7%) was identified as the second most common serovar in chicken/chicken products. However, unlike *S.* Enteritidis, it was not often associated with human illness in Singapore. *S*. Heidelberg was predominantly found in frozen chicken meat, whereas *S*. Enteritidis was found in both fresh and frozen chicken meat. *S.* Heidelberg was one of the main serovars implicated in large multistate outbreaks in humans in the United States, reportedly associated with the consumption of contaminated poultry and poultry products [7]. In South America, an increased occurrence of *S.* Heidelberg in poultry slaughterhouses was reported by the Brazilian government control programs [23]. In line with this, the European Commission also reported the detection of *S.* Heidelberg in frozen chicken liver imported from Brazil [24].

Chilled/fresh chicken may be preferred over frozen chicken by the Singaporean population, and dietary preference may explain in part the phenomenon that *S.* Heidelberg was not usually associated with human illness in Singapore from 2012 to 2016. This further underline the necessity of assessing the risk of salmonellosis associated with different food commodities. Quantitative microbial risk assessment can be applied to model the exposure and probability of salmonellosis associated with consumption of poultry commodities, for strategizing of mitigation measures to reduce *Salmonella* infection burden [25].

Serovar 4,5,12:b:-(dT+) was one of the most common serovars associated with human salmonellosis over the 5-year study period, but did not appear as a common serovar in all food and animal-related sample categories in this study. Serovar 4,5,12:b:-(dT+) is a *d*-tartrate fermenting variant of *S.* Paratyphi B which can cause gastroenteritis in humans [26]. It has been reported that *Salmonella* isolates belonging to 4,5,12:b:-(dT+) are genetically highly diverse. The serovar has been isolated from a wide variety of sources including poultry, reptiles, fish, mushrooms and turtles [27]. Two monophasic strains belonging to the MLST sequence types ST42 and ST423 were isolated in Singapore from local wild birds (black bittern and crow) in 2012 [28]. In 2018, the serovar 4,5,12:b:-(dT+) was reported to be responsible for a multistate outbreak of *Salmonella* infection due to consumption of products containing Kratom, a tropical plant native to Southeast Asia [29]. In Germany, the serovar contributed to 0.02% (96/50, 705) of human salmonellosis, with potential sources identified to be most likely mushrooms–with linkage to an import from Asia, and fish/shellfish [27]. In this study, the monophasic serovar 4,5,12:b:-(dT+) was not detected among food and animal isolates. Serovar 4,5,12:b:-(dT+) was reported to be polyphyletic and could be identified as *S.* Paratyphi B or *S.* Abony [27]. It was noted that neither *S.* Paratyphi B nor *S.* Abony was detected among the food and animal isolates as a frequent serovar. We propose further studies on serovar 4,5,12:b:-(dT+), to investigate if this serovar may have originated from other sources not covered in this study.

*S.* Stanley was the second most prevalent serovar associated with human cases in the 2012–2014 period. *S.* Stanley is endemic to Asia and infections are frequently associated with travel to Southeast Asian countries [30]. Other non-travel-related outbreaks have been linked to contaminated alfalfa sprouts, peanuts, soft cheese and turkey in Europe [31,32,33,34]. In this study, *S.* Stanley was identified in nearly all food categories (chicken, duck, other poultry, pork, beef, mutton and other meats) in varying detection rates (1.3–7.9%). It was the most common species detected in frog, crocodile and turtle meat samples. However, the sample size of the food sub-category was relatively small. With the current limited evidence, it is difficult to draw a conclusion on the principal vehicle of *S.* Stanley. In addition to foods, wild birds residing in the local environment were previously identified as reservoirs for *S*. Stanley. In Singapore’s endeavors towards urban rewilding, assimilation of nature elements into the city promotes closer proximity between wildlife and human habitats which could possibly play a role in the local epidemiology of zoonosis and reverse zoonosis [28]. Further studies complemented with whole genome sequencing may allow better epidemiologic insights into human acquisition of diseasing-causing *S.* Stanley in Singapore, and subsequent mitigation of the public health risk of transmission from potential reservoirs to humans.

*S.* Weltevreden was identified as the third-most prevalent serovar among human isolates. The serovar was the most frequently isolated in seafood in this study, contributing to approximately 20.0% of all seafood isolates from 2012 to 2016. Fish and seafood consumption by the population is relatively high (21 kg per capita per annual) and cooking methods may vary (raw consumption or to avoid overcooking seafood), allowing the survival of bacteria. *S.* Weltevreden is increasingly associated with human infections and outbreaks in Southeast Asia where it appears to be an emerging foodborne pathogen [35,36,37]. It is known to be wide-spread in water-related environments, possibly due to its ability to persist and multiply in tropical aquatic environments [9,38,39,40]. In Singapore, *S.* Weltevreden was also isolated from retail cooked or ready-to-eat food as well as from wild birds, suggesting its ability to survive and colonize in different types of host and environment [28]. An increased and targeted sampling of different types of seafood and risk assessment studies complemented with molecular epidemiological comparison studies of human *S*. Weltevreden isolates are recommended to identify main seafood types associated with human *S*. Weltevreden infections.

Two other serovars, *S.* Brancaster and *S.* Saintpaul, which recently appeared among the top 10 most prevalent serovars in humans and foods in 2015 and 2016, were primarily found in fresh chicken meat. In 2018, a study in Singapore reported that *S.* Brancaster (21.2%) and *S.* Saintpaul (32.7%) were among the most prevalent serovars found in fresh retail chicken meat in markets and supermarkets [8]. Similarly, *S*. Brancaster was identified as one of the three predominant serovars in poultry meat collected from slaughterhouses, small-scale poultry processing plants and wet markets in Malaysia from 2013 to 2017 [41,42]. In the same country, *S.* Saintpaul was reported to be recovered from indigenous vegetables including Vietnamese coriander and water spinach [43,44]. *S*. Saintpaul was one of the serovars contributing to more than half of the fresh produce-related outbreaks associated with fruits, vegetables and sprouts from 2001 to 2016 in Australia [45]. These previous reports suggest that, compared to other food categories, *S.* Saintpaul could more commonly be associated with fresh produce which is usually consumed raw and is a food category not routinely covered in the surveillance programs included in this study. The recent findings of *S.* Brancaster and *S.* Saintpaul in clinical cases highlight the need for continuous close monitoring of the distribution of these serovars in potential sources, e.g., poultry and fresh produce, which are possible food vehicles contributing to the increasing incidence of human salmonellosis.

Among *Salmonella* isolates found in pork and pork products, *S.* Derby was the second most frequent serovar after *S.* Typhimurium, accounting for nearly 20.0% of all isolates in this food category. In Europe and the United States, *S.* Derby was the most abundant serovar isolated from pork and was also frequently isolated from human clinical samples [46]. It can colonize and persist in swine populations and pig production environments and has been reportedly linked to several human salmonellosis outbreaks [46,47,48,49]. On the contrary, like *S.* Heidelberg, *S.* Derby was not often associated with human salmonellosis and did not appear to be a major public health concern in Singapore at the time the study was being carried out.

This study presents One-Health agencies’ combined retrospective data on *Salmonella* in foods and animals as a step towards identifying factors potentially contributing to human salmonellosis in Singapore. The study was however limited by several factors. Firstly, data were extracted from various surveillance programs which might be subjected to inherent sampling bias. For instance, food samples were collected on a risk-based schedule, taking into consideration factors such as risk category, compliance history, origin of source and importers’ track records, and additional sampling of the ‘problem source’ would be triggered upon initial detection, which might result in an inherently biased sampling. Furthermore, clinical isolates included in the study were from selected public hospitals and might not represent the complete case data. It should also be noted that human isolates associated with outbreak cases might affect the serovar estimation analysis due to close relatedness of the outbreak isolates. Secondly, the screening of specific *Salmonella* serovars was dependent on surveillance program. For instance, imported shell eggs and veterinary samples were primarily screened for *S.* Enteritidis and *S.* Typhimurium which were the target serovars of the respective national surveillance programs. Hence, this might limit the potential to estimate the full-range diversity of all possible serovars and therefore, information on the diversity of serovars in these samples might be less complete than that of other sample categories. Thirdly, sample sizes in certain food product sub-categories such as frog, crocodile, turtle, beef and mutton were limited for inference drawing about trend over the study period. Lastly, the conventional serotyping method might have limited discriminatory power which prevented further delineation between isolates of the same serovar. For instance, re-sampling at the same farm or premise as part of monitoring control may result in collection of isolates originating from the same source which could plausibly lead to overestimation/underestimation of serovars. An alternative typing method with higher resolution such as whole genome sequencing may be further applied for assessment of genetic relatedness between isolates of the same serovar from the same isolation source. These gaps are well-recognized and currently being reviewed to enhance *Salmonella* surveillance programs in various sectors.

## 5. Conclusions

This is the first study that collectively describes the distribution of *Salmonella* serovars in humans, foods, and animals in a One-Health approach for enhanced understanding of the epidemiology of human salmonellosis in Singapore. *S.* Enteritidis was predominantly isolated from human cases. The same serovar was the most frequently identified serovar in poultry/poultry products and eggs/egg products. Findings also revealed the distribution of *Salmonella* serovars and possible various transmission pathways associated with humans are proposed. Findings from this study reiterate the importance of good hygiene and food preparation practices to limit the spread of *Salmonella* from potential reservoirs as well as the need for continuous monitoring. Finally, with the joint effort of health protection framework involving One-Health, this study provides useful insights for designing and implementing more effective and integrated surveillance systems for *Salmonella*.

## Figures and Tables

**Table 1:** Number of *Salmonella* isolates included in this study (2012–2016).

Sample Category	*n*	%
** Human—clinical**	4462	-
** Food**	2529	-
Chicken and chicken products	1538	60.8
Pork and pork products	421	16.6
Duck	223	8.8
Seafood	120	4.7
Other poultry	100	4.0
Frog, crocodile and turtle	63	2.5
Beef and mutton	38	1.5
Eggs and egg products	26	1.0
** Farms**		
Chicken and quail layers	945	-
** Companion and wildlife animals**	68	-

**Table 2:** Frequency of *Salmonella* serovars in humans, farms, foods, companion and wildlife animal samples in Singapore from 2012 to 2016.

Sample Source		2012			2013			2014			2015			2016		
	Rank	Serovar	n	%	Serovar	n	%	Serovar	n	%	Serovar	n	%	Serovar	n	%
**Humans**	1	Enteritidis	161	21.8	Enteritidis	271	32.0	Enteritidis	269	27.6	Enteritidis	200	22.2	Enteritidis	251	25.0
	2	Stanley	91	12.3	Stanley	107	12.6	Stanley	151	15.5	Weltevreden	100	11.1	Weltevreden	104	10.4
	3	Weltevreden	81	11.0	Weltevreden	73	8.6	Weltevreden	97	9.9	Saintpaul	96	10.7	Stanley	100	10.0
	4	4,5,12:b:(dT+)	47	6.4	Typhi	56	6.6	Typhimurium	72	7.4	Stanley	80	8.9	Saintpaul	80	8.0
	5	Typhimurium	42	5.7	4,5,12:b:(dT+)	52	6.1	4,5,12:b:(dT+)	62	6.4	4,5,12:b:(dT+)	49	5.4	4,5,12:b:(dT+)	60	6.0
	6	Typhi	36	4.9	Typhimurium	44	5.2	Albany	34	3.5	Typhi	38	4.2	Typhi	47	4.7
	7	Bareilly	32	4.3	Bareilly	26	3.1	Typhi	33	3.4	Typhimurium	36	4.0	Bareilly	35	3.5
	8	Albany	23	3.1	Javiana	22	2.6	Bareilly	23	2.4	Paratyphi B Var Java	28	3.1	Hvittingfoss	31	3.1
	9	Paratyphi A	20	2.7	Paratyphi B var Java	18	2.1	Javiana	22	2.3	Albany	24	2.7	Typhimurium	29	2.9
	10	Javiana	19	2.6	Braenderup	17	2.0	Hvittingfoss	22	2.3	Bareilly	23	2.6	Brancaster	25	2.5
		Others	185	25.1	Others	161	19.0	Others	190	19.5	Others	227	25.2	Others	240	24.0
		Total	737		Total	847		Total	975		Total	901		Total	1002	
**Foods**	**Rank**	**Serovar**	**n**	**%**	**Serovar**	**n**	**%**	**Serovar**	**n**	**%**	**Serovar**	**n**	**%**	**Serovar**	**n**	**%**
	1	Typhimurium	135	22.8	Typhimurium	95	18.9	Typhimurium	54	15.2	Brancaster	65	14.8	Brancaster	32	12.3
	2	Heidelberg	132	22.3	Enteritidis	91	18.1	Albany	39	11.0	Albany	65	14.8	Typhimurium	30	11.5
	3	Enteritidis	95	16.0	Heidelberg	68	13.5	Enteritidis	34	9.6	Enteritidis	60	13.7	Heidelberg	22	8.4
	4	Albany	34	5.7	Minnesota	31	6.2	Heidelberg	29	8.1	Typhimurium	27	6.2	Enteritidis	20	7.7
	5	Stanley	24	4.0	Albany	30	6.0	Minnesota	23	6.5	Mbandaka	24	5.5	Stanley	19	7.3
	6	Kentucky	23	3.9	Stanley	26	5.2	Braenderup	16	4.5	Heidelberg	15	3.4	Saintpaul	14	5.4
	7	Anatum/ var 15	12	2.0	Infantis	24	4.8	Corvallis	13	3.7	Corvallis	14	3.2	Mbandaka	10	3.8
	8	Weltevreden	11	1.9	Mbandaka	21	4.2	Istanbul	12	3.4	Saintpaul	12	2.7	Potsdam	9	3.4
	9	Schwarzengrund	10	1.7	Weltevreden/var15	10	2.0	Kentucky	12	3.4	Stanley	11	2.5	Weltevreden/var15	9	3.4
	10	Derby	10	1.7	Braenderup	9	1.8	Derby	11	3.1	Derby	9	2.1	Derby	6	2.3
		Others	107	18.0	Others	98	19.5	Others	113	31.7	Others	136	31.1	Others	90	34.5
		Total	593		Total	503		Total	356		Total	438		Total	261	
**Chicken layer farms**	**Rank**	**Serovar**	**n**	**%**	**Serovar**	**n**	**%**	**Serovar**	**n**	**%**	**Serovar**	**n**	**%**	**Serovar**	**n**	**%**
	1	Enteritidis	4	4.7	Enteritidis	8	6.7	Enteritidis	71	24.4	Enteritidis	7	6.0	Enteritidis	9	21.4
	2	Others	82	95.3	Typhimurium	8	6.7	Typhimurium	12	4.1	Typhimurium	2	1.7	Others	33	78.6
					Others	103	86.6	Others	208	71.5	Others	107	92.2			
		Total	86		Total	119		Total	291		Total	116		Total	42	
**Quail layer farms**	**Rank**	**Serovar**	**n**	**%**	**Serovar**	**n**	**%**	**Serovar**	**n**	**%**	**Serovar**	**n**	**%**	**Serovar**	**n**	**%**
	1	Typhimurium	15	20.5	Typhimurium	11	11.5	Typhimurium	4	6.3	Typhimurium	5	10.6	Typhimurium	2	16.7
	2	Others	58	79.5	Others	85	88.5	Others	59	93.7	Others	42	89.4	Typhimurium (monophasic)	1	8.3
														Others	9	75.0
		Total	73		Total	96		Total	63		Total	47		Total	12	
**Companion and wildlife animals**	**Rank**	**Serovar**	**n**	**%**	**Serovar**	**n**	**%**	**Serovar**	**n**	**%**	**Serovar**	**n**	**%**	**Serovar**	**n**	**%**
	1	Typhimurium	1	5.6	Typhimurium	2	14.3	Typhimurium	0	0.0	Typhimurium	0	0.0	Typhimurium	1	7.1
	2	Enteritidis	0	0.0	Enteritidis	1	7.1	Enteritidis	0	0.0	Enteritidis	0	0.0	Enteritidis	0	0.0
		Others	17	94.4	Others	11	78.6	Others	10	100.0	Others	12	100.0	Others	13	92.9
		Total	18		Total	14		Total	10		Total	12		Total	14	

The *n* represents the number of isolates related to each serovar, whereas % represents the relative occurrence of serovars within the sample category.

**Table 3:** Frequency of *Salmonella* serovars in various sample categories (2012–2016).

**Rank**	**Human—Clinical**			**Chicken and Chicken Products**			**Pork and Pork Products**		
	**Serovar**	**n**	**%**	**Serovar**	**n**	**%**	**Serovar**	**n**	**%**
1	Enteritidis	1152	25.8	Enteritidis	438	28.5	Typhimurium	148	35.2
2	Stanley	529	11.9	Heidelberg	333	21.7	Derby	79	18.8
3	Weltevreden	455	10.2	Typhimurium	145	9.4	Infantis	22	5.2
4	4,5,12:b:(dT+)	270	6.1	Albany	103	6.7	Stanley	18	4.3
5	Typhimurium	223	5.0	Minnesota	80	5.2	Anatum/var	7	1.7
6	Typhi	210	4.7	Kentucky	60	3.9	Saintpaul	6	1.4
7	Saintpaul	176	3.9	Corvallis	42	2.7	Agona	5	1.2
8	Bareilly	139	3.1	Stanley	40	2.6	Bovismorbificans	5	1.2
9	Albany	81	1.8	Schwarzengrund	37	2.4	Minnesota	3	0.7
10	Javiana	63	1.4	Braenderup	28	1.8	Braenderup	3	0.7
	Other	1164	26.1	Other	232	15.0	Other	125	29.7
	Total	4462		Total	1538		Total	421	
**Rank**	**Duck**			**Seafood**			**Other poultry (quail, turkey)**		
	**Serovar**	**n**	**%**	**Serovar**	**n**	**%**	**Serovar**	**n**	**%**
1	Typhimurium	98	43.9	Weltevreden/var15+	23	19.2	Typhimurium	21	21.0
2	Hadar	20	9.0	Typhimurium	13	10.8	Infantis	13	13.0
3	Enteritidis	17	7.6	Brancaster	6	5.0	Agona	4	4.0
4	Anatum/var	9	4.0	Stanley	4	3.3	Weltevreden/var15+	3	3.0
5	Kentucky	5	2.2	Mbandaka	4	3.3	Heidelberg	2	2.0
6	Infantis	5	2.2	Albany	2	1.7	Stanley	2	2.0
7	Albany	4	1.8	Corvallis	2	1.7	Corvallis	2	2.0
8	Corvallis	4	1.8	Braenderup	2	1.7	Enteritidis	1	1.0
9	Weltevreden/var15+	4	1.8	Bovismorbificans	2	1.7	Mbandaka	1	1.0
10	Stanley	3	1.3	Enteritidis	1	0.8	Hadar	1	1.0
	Other	54	24.2	Other	61	50.8	Other	50	50.0
	Total	223		Total	120		Total	100	
**Rank**	**Other meat** **(frog, crocodile and turtle)**			**Beef and mutton**			**Eggs and egg products**		
	**Serovar**	**n**	**%**	**Serovar**	**n**	**%**	**Serovar**	**n**	**%**
1	Stanley	5	7.9	Typhimurium	6	15.8	Enteritidis	16	61.5
2	Braenderup	4	6.3	Bovismorbificans	5	13.2	Typhimurium	4	15.4
3	Typhimurium	3	4.8	Infantis	4	10.5	Braenderup	2	7.7
4	Corvallis	2	3.2	Stanley	2	5.3	Mbandaka	1	3.8
5	Anatum/var	2	3.2	Mbandaka	2	5.3	Other	3	11.5
6	Albany	1	1.6	Other	19	50.0			
	Other	46	73.0						
	Total	63		Total	38		Total	26	
**Rank**	**Chicken and quail layer farms**			**Companion and wildlife animals**					
	**Serovar**	**n**	**%**	**Serovar**	**n**	**%**			
1	Enteritidis	99	10.5	Typhimurium	4	5.9			
2	Typhimurium	59	6.2	Enteritidis	1	1.5			
3	Typhimurium (monophasic)	1	0.1	Other	63	92.6			
	Other	786	83.2						
	Total	945		Total	68				

The *n* represents the number of isolates related to each serovar, whereas % represents the relative occurrence of serovars within the sample category.

**Table 4:** Top five most common *Salmonella* serovars found in chicken samples from 2012 to 2016.

Sample Source		2012			2013			2014			2015			2016		
	Rank	Serovar	n	%	Serovar	n	%	Serovar	n	%	Serovar	n	%	Serovar	n	%
**Fresh chicken**	1	Enteritidis	16	29.1	Enteritidis	42	55.3	Albany	7	21.9	Albany	8	21.6	Brancaster	9	27.3
	2	Albany	15	27.3	Albany	17	22.4	Corvallis	7	21.9	Brancaster	6	16.2	Saintpaul	9	27.3
	3	Typhimurium	8	14.5	Braenderup	5	6.6	Typhimurium	4	12.5	Enteritidis	5	13.5	Typhimurium	4	12.1
	4	Stanley	7	12.7	Typhimurium	4	5.3	Enteritidis	3	9.4	Corvallis	5	13.5	Enteritidis	4	12.1
	5	Corvallis	2	3.6	Stanley	4	5.3	Braenderup	3	9.4	Saintpaul	3	8.1	Mbandaka	2	6.1
		Others	7	12.7	Others	4	5.3	Others	8	25.0	Others	10	27.0	Others	5	15.2
		Total	55		Total	76		Total	32		Total	37		Total	33	
**Frozen chicken**	**Rank**	**Serovar**	**n**	**%**	**Serovar**	**n**	**%**	**Serovar**	**n**	**%**	**Serovar**	**n**	**%**	**Serovar**	**n**	**%**
	1	Heidelberg	128	48.9	Heidelberg	68	49.6	Heidelberg	29	25.9	Enteritidis	46	34.8	Heidelberg	22	47.8
	2	Typhimurium	42	16.0	Minnesota	31	22.6	Minnesota	20	17.9	Albany	29	22.0	Enteritidis	3	6.5
	3	Enteritidis	39	14.9	Enteritidis	11	8.0	Enteritidis	15	13.4	Heidelberg	15	11.4	Schwarzengrund	3	6.5
	4	Kentucky	20	7.6	Infantis	5	3.6	Albany	9	8.0	Virchow	9	6.8	Stanley	3	6.5
	5	Schwarzengrund	7	2.7	Schwarzengrund	4	2.9	Braenderup	7	6.3	Liverpool	5	3.8	Kentucky	2	4.3
		Others	26	9.9	Others	18	13.1	Others	32	28.6	Others	28	21.2	Others	13	28.3
		Total	262		Total	137		Total	112		Total	132		Total	46	

The *n* represents the number of isolates related to each serovar, whereas % represents the relative occurrence of serovars within the sample category.
